# The Use of Twitter by Medical Journals: Systematic Review of the Literature

**DOI:** 10.2196/26378

**Published:** 2021-07-28

**Authors:** Natalie Erskine, Sharief Hendricks

**Affiliations:** 1 Division of Physiological Sciences Department of Human Biology Faculty of Health Sciences, University of Cape Town Cape Town South Africa; 2 Health, Physical Activity, Lifestyle, and Sport Research Centre Department of Human Biology, Faculty of Health Sciences University of Cape Town Cape Town South Africa; 3 Carnegie Applied Rugby Research Centre Institute for Sport, Physical Activity and Leisure Leeds Beckett University Leeds United Kingdom

**Keywords:** Twitter, social media, medical journals, impact

## Abstract

**Background:**

Medical journals use Twitter to engage and disseminate their research articles and implement a range of strategies to maximize reach and impact.

**Objective:**

This study aims to systematically review the literature to synthesize and describe the different Twitter strategies used by medical journals and their effectiveness on journal impact and readership metrics.

**Methods:**

A systematic search of the literature before February 2020 in four electronic databases (PubMed, Web of Science, Scopus, and ScienceDirect) was conducted. Articles were reviewed using the PRISMA (Preferred Reporting Items for Systematic Reviews and Meta-analyses) guidelines.

**Results:**

The search identified 44 original research studies that evaluated Twitter strategies implemented by medical journals and analyzed the relationship between Twitter metrics and alternative and citation-based metrics. The key findings suggest that promoting publications on Twitter improves citation-based and alternative metrics for academic medical journals. Moreover, implementing different Twitter strategies maximizes the amount of attention that publications and journals receive. The four key Twitter strategies implemented by many medical journals are tweeting the title and link of the article, infographics, podcasts, and hosting monthly internet-based journal clubs. Each strategy was successful in promoting the publications. However, different metrics were used to measure success.

**Conclusions:**

Four key Twitter strategies are implemented by medical journals: tweeting the title and link of the article, infographics, podcasts, and hosting monthly internet-based journal clubs. In this review, each strategy was successful in promoting publications but used different metrics to measure success. Thus, it is difficult to conclude which strategy is most effective. In addition, the four strategies have different costs and effects on dissemination and readership. We recommend that journals and researchers incorporate a combination of Twitter strategies to maximize research impact and capture audiences with a variety of learning methods.

## Introduction

The main goals of health science research are to improve health, services, and practice, as well as develop health care technologies [1]. Research needs to be translated from *what we know* to *what we do* to achieve these goals [1]. This process is generally referred to as knowledge translation [2]. Although knowledge translation does not have an agreed-upon definition [3], it typically includes all the steps from the creation of new knowledge to its application [4]. One primary step in knowledge translation is dissemination, that is, the communication and sharing of research findings [1]. Medical journals are a key source of information for health researchers and practitioners. They also play an important role in dissemination [5]. Traditional journal dissemination is passive, unplanned, and uncontrolled [1] and relies on the end user to search for information (known as the *pull* concept). This approach requires awareness of and access to the journal, which is not always the case for health researchers and practitioners [6].

Twitter is a microblogging service that allows the sharing of short messages (tweets) within a 280-character limit and can include images, videos, and hyperlinks to other sites. Users can share the tweet (retweet) with their web-based network or community (followers) [7].

Twitter’s global popularity and ubiquitous nature offers a rapid, accessible, and cost-effective medium for communication and sharing of information [8]. Among health professionals and researchers, Twitter is increasingly being used as a medium for health communication to stakeholders [9]. Medical journals have also recognized the potential of Twitter to disseminate their research articles and have implemented a range of strategies to maximize reach [10]. The simplest strategy is posting the title of the research article with a link to the article on the journal’s website. Beyond this, medical journals have also summarized research findings into a single post; produced infographics, visual abstracts, and podcasts; and hosted Twitter discussions and journal clubs [10]. In contrast to traditional dissemination, this approach can be considered active, as the journal *pushes* content to its audience [1].

Increased dissemination of research on Twitter can improve article readership and the impact of a journal in terms of citation-based and alternative metrics [10], such as impact factors [11] and citation counts [12]. Research dissemination on Twitter is measured using alternative metrics (*Altmetrics*) and includes Altmetric attention scores, pageviews, article downloads, and Twitter metrics. Altmetrics measure how often an article has been shared, viewed, or referenced on the web by both professional and lay audiences [13]. Compared with citation-based metrics, Altmetrics provide detailed and real-time feedback on the web-based reach and impact of a research article [13]. Despite all the professional benefits it has to offer, Twitter is still an underused tool among medical journals, with less than a third hosting a Twitter profile [14]. One of the main reasons for this underutilization is the lack of evidence-based best practices [10,15]. Thus, studies have been conducted on the effects of different Twitter strategies on article dissemination and journal impact. However, this research has not been synthesized in a meaningful way to inform practice. Therefore, the purpose of this review is to synthesize and describe the different Twitter strategies used by medical journals and their effectiveness on article dissemination, readership, and journal impact metrics.

## Methods

### Overview

For this review, Twitter was defined as *“*a microblogging and social networking service on which users post and interact with messages.” Medical journals were defined as *“*a peer-reviewed scientific journal that communicates medical information to health practitioners.” Journal impact was measured using citation-based metrics, such as citation count and impact factor. Article readership was measured using pageviews and full-text article downloads. Article dissemination was measured using Altmetric attention scores and Twitter metrics, including impressions, engagements, link clicks, and retweets. The definitions of each dissemination metric are provided in Textbox 1.

**Definitions of outcome measures reported by included studies.**

**Retweets**

- The number of times a user retweeted (reposted) a tweet. This feature allows users to share information from the source with their followers. You can retweet your Tweets or Tweets from someone else [16].

**Impressions**

- The number of people who saw your tweet [17].

**Engagements**

- The total number of times a user interacted with a Tweet. Clicks anywhere on the Tweet, including retweets, replies, follows likes, links, cards, hashtags, embedded media, username, profile photo, or Tweet expansion [17].

**Link Clicks**

- The number of times the article link in the Tweet is clicked [17].

**Downloads**

- The number of times a research article was download as a pdf from the website of the medical journal [18].

**Pageviews**

- The number of times an article on the website of the medical journal was loaded on a web browser (abstract and full text combined) irrespective of the source [18].

**Altmetric Score**

- A weighted count of all the web-based attention an individual research output has received from web-based media platforms, including social media networks, news outlets, blogs, and others [19].

### Literature Search Strategy

A systematic review was conducted to retrieve all relevant research studies. All study designs were included in the review to identify the best evidence available to address the research objectives. The literature search was conducted using the following four electronic databases: PubMed, Web of Science, Scopus, and ScienceDirect. The search was performed using the following search terms: “MEDICAL,” “MEDICINE,” “JOURNALS,” “SOCIAL MEDIA,” and “TWITTER.” For example, we searched PubMed using the following strategy: (“medical” OR “medicine”) AND “journals” AND “social media” AND “Twitter.”

### Article Selection

Following the collection of studies from different electronic databases, duplicate studies were removed and screened for eligibility using the PRISMA (Preferred Reporting Items for Systematic Reviews and Meta-Analyses) guidelines. The inclusion criteria were as follows: (1) English language, full-text original research articles; (2) studies of medical journals; (3) evaluations of Twitter strategies implemented for article dissemination (eg, infographics and web-based journal clubs) or analysis of the relationship between alternative metrics and citation-based metrics; (4) published any time before February 2020; and (5) all research study designs. The exclusion criteria were (1) studies not in English; (2) literature reviews, dissertation theses, review papers, reports, conference papers or abstracts, letters to the editor, commentaries, and feature articles; and (3) studies involving nonmedical academic journals. Following abstract and title screening, we conducted a full-text review of the selected articles.

### Data Extraction, Synthesis, and Evaluation

The following data were extracted from the included studies: first author, year of publication, study aim, study sample, Twitter strategy implemented, study methodology (description of the Twitter strategy implemented or metrics being analyzed), primary study outcomes, and results. A customized data extraction sheet was developed using Microsoft Excel. The Medical Education Research Study Quality Instrument was used to evaluate the quality of all included studies [20].

## Results

### Overview

**Figure 1:**
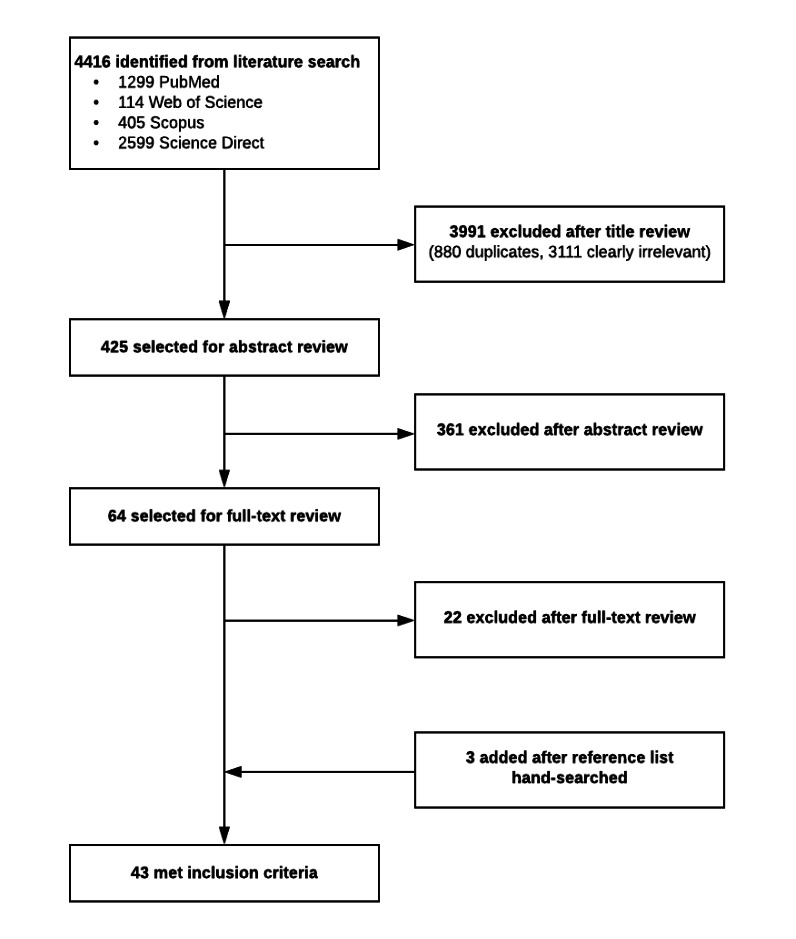
Preferred Reporting Items for Systematic Reviews and Meta-analyses flow chart for search strategy.

The database search identified 4416 titles (Figure 1). After removing the duplicates and reviewing the titles, 425 articles remained for abstract review. After reviewing the abstracts, we retrieved 64 full-text articles. The reference list of the 64 full-text articles were manually searched. The total number of included studies was 44. Multimedia Appendix 1 provides a summary of the included studies.

### Characteristics of Included Studies

Of the 43 included studies, 27 (63%) health-related disciplines were reviewed, with urology (6/43, 14%) and radiology (5/43, 9%) representing the top two disciplines. A total of 74% (32/43) of the published studies were within the last 4 years. A total of 53% (23/43) of the studies analyzed the relationship between alternative and citation-based metrics as their primary objective. The remaining 47% (20/43) studied how specific Twitter strategies used by medical journals impacted these relationships. In all, 74% (32/43) of studies used an observational study design [12,14,21-43,45-50] and 26% (11/43) used an experimental design [11,18,44,51-58]. Multimedia Appendix 1 presents the key findings of each study.

### Assessing the Quality of Studies

The studies received an average score of 9.5 out of 13.5, ranging from 8 to 10.5. A total of 70% (30/43) of the studies scored above 9.5. For detailed scores, see Multimedia Appendix 2 [10-14,18,21-54,56-59].

### Journals With a Twitter Account Have Greater Citation-Based and Alternative Metric Scores

Studies that compared journals with and without a Twitter account showed that Twitter has a higher impact factor (n=4) [27,29,32,34], an increase in Altmetric attention scores (n=1) [39], H-index scores (n=3) [21,31,35], SCImago Journal Rank (n=4) [21,31,35,40], and receive more citations (n=1) and tweets [12].

### Number of Tweets May Be Positively Correlated to Publication Citations

A total of 6 studies [22,25,26,28,37,38] reported a positive and significant relationship between Twitter mentions and article citations, whereas 2 studies reported no significant relationship [57,58].

### Implementing Twitter Strategies Increases Research Dissemination

Twitter strategies implemented by medical journals to promote their research articles ranged from hosting internet-based journal clubs (7/43, 16%) [42-44,46-50], standard article promotion (7/43, 16%) [11,44,52,53,55,57,58], infographics or visual abstracts (6/43, 14%) [18,45,50,51,54,56], and podcasts (1/43, 2%) [56]. The effects of each strategy are shown in Multimedia Appendix 1, and the definitions for each strategy are provided in Textbox 2. The primary outcome measures included impressions, engagements, link clicks, pageviews (full-text or abstract or both), full-text downloads, and Altmetric scores. The definitions of each outcome are presented in Textbox 1.

**Definitions of the Twitter strategies implemented by journals.**

**Journal Club**

- Participants discuss a selected research article virtually using Twitter, often meeting at a set date and time.

**Basic Tweet**

- Posting a message on Twitter with the title of an academic article along with a link to the full-text version on the journals’ websites. The article may or may not be accessible.

**Infographic**

- Posting a message on Twitter with the title of an academic article, a summary of the results in the form of an infographic, and a link to the full-text version on the journals’ websites. The article may or may not be accessible.

**Podcast**

- Posting a message on Twitter containing the title of an academic article and a link to the full-text version of the article as well as a downloadable podcast based on the article on the journals’ websites. The article may or may not be accessible.

**Observational**

- No intervention was implemented. The authors analyzed the relationship between Twitter metrics or alternative metrics and citation-based metrics for journals or articles.

## Discussion

### Principal Findings

This is the first review to analyze the use of Twitter in medical journals and its effect on research dissemination, readership, and journal impact metrics. The first key finding of this review is that journals should endeavor to have a dedicated Twitter account to promote their publications. This Twitter promotion will improve article dissemination, article readership, and citation-based metrics, ultimately leading to an increase in the impact factor. Beyond this, implementing different Twitter strategies maximizes the web-based dissemination of and engagement with publications and journals. There were four main Twitter strategies implemented by medical journals: basic tweeting, infographics, podcasts, and internet-based Twitter journal clubs. Each strategy was successful in the dissemination of publications, but different metrics were used to measure success. Thus, it was difficult to conclude which strategy was the most effective. The discussion below details the benefits and challenges of each strategy and how they increase the potential for successful dissemination.

### Basic Tweeting

Basic tweeting was the simplest Twitter strategy used by medical journals, that is, tweeting the title of the article along with the link to the abstract or full-text version on the journal’s website. Basic tweeting had varying impacts on article readership and dissemination, based on a number of factors. These factors include the source of the tweet, posting frequency, and the number of Twitter followers.

The tweet source is as important as the content of the tweet. For example, Hawkins et al [44] increased weekly pageviews per article by 139% when the editorial board tweeted articles from their personal accounts compared with articles tweeted from the journal account only. In another study, using a pre-post study design on Twitter, a journal website received a 273% increase in monthly pageviews after a team of physicians and medical graduates (who were active social media users) began tweeting articles from their personal accounts [11]. Similarly, Luc et al [55] showed that tweeting journal articles via the personal accounts of key opinion leaders significantly increased 7-day posttweet Altmetric scores, Mendeley reads, and Twitter impressions. On the basis of these results, we recommend that journals should encourage key opinion leaders, editorial board members, and active social media users in their medical disciplines to promote articles on their personal Twitter accounts on behalf of the journal.

Increasing the posting frequency may increase the dissemination of articles. Fox et al [52] found no increase in pageviews for articles posted only twice. In contrast, Widmer et al [58] found a 900% increase in pageviews via Twitter for articles posted seven times. In another study, Hawkins et al [44] found that 4 tweets per article resulted in significantly more pageviews than 1 tweet per article. Although the studies differed in design, collectively, these results suggest that posting frequency plays an important role in the effectiveness of the basic tweet strategy and that a dose-response relationship between tweeting and impact may exist [58].

A large number of Twitter followers are essential for the success of social media promotion. Two studies, both by Fox et al [52,53], found no increase in monthly article pageviews after implementing a low-intensity (two posts per article) and high-intensity (three posts per article) posting frequency on the journal’s Twitter account. The journal accounts only had 2219 and 10,072 Twitter followers for each study. In contrast, Luc et al [55] and Widmer et al [58] had 52,983 and 1,177,514 Twitter followers, respectively, at the start of their intervention. From a social media recommendation for the journals’ perspective, this means journals starting out on Twitter should focus on increasing followers as opposed to being overly concerned about readership metrics.

Basic article promotion is an easy, cost-effective, and time-effective strategy for journals. From a reader’s perspective, it keeps them up to date with the latest publications, especially if they choose to be notified every time a journal tweets. However, it requires readers to click the link to the journal’s website to read the abstract of the article [51]. In addition, nonscientific readers may not be able to read paid-access articles and may perceive a text abstract as confusing and boring. Clicking the link, however, is beneficial for the journal as it increases their web traffic.

### Visual Abstracts

Including a visual abstract, that is, a simplified graphical summary of a study’s scientific abstract, within article tweets increases their dissemination on Twitter and, subsequently, readership. In contrast to basic tweets, the visual abstract tweets receive significantly more abstract pageviews, impressions [45,51,54], engagements [45,51], and Altmetric attention scores [56]. However, visual abstracts enhance the impact of basic tweets, which is referred to as the *spillover effect*. For example, two studies reported an increase in impressions, pageviews and engagements for basic tweets sent from the same journal account after implementing the visual abstract strategy [45,54].

Thoma et al [56] and Huang et al [18] found that visual abstracts did not increase full-text readership. Huang et al [18] provided two suggestions for this finding. First, readers may have felt that the visual abstract was a sufficient summary and did not need to read the full-text article [18]. This suggestion justifies the lower number of link clicks received for visual abstract tweets than for basic tweets. Second, readers may not have had access to full-text articles [18]. If journals choose to provide visual abstracts, they need to ensure that the information presented is accurate and easily digestible to prevent misinterpretation and the spread of misinformation.

The responsibility of designing visual abstracts is usually left to the journal editors, which is costly and time-consuming, even though open-source templates are available to help authors create their own [45]. The positive impact of visual abstracts provides a motive for the feasibility of this strategy and for journals to acquire the required resources. Visual abstracts provide an engaging and digestible summary of the research, which is beneficial to nonscientific readers who may not have access to full-text articles. As such, we recommend that journals allocate resources to the creation of visual abstracts.

### Podcast

Articles tweeted with linked podcasts receive greater increases in Altmetric attention scores and abstract pageviews than visual abstracts or basic tweets, although they show no increase in full-text readership [56]. A possible explanation for this is that during the podcast, the research paper is critically analyzed and discussed, eliminating the need for users to read the full publication themselves [60]. Podcasts are convenient and accessible and can be heard while performing other tasks, such as commuting, household tasks, or exercise [61]. Podcasts are also enjoyable to listen to due to their entertainment and educational value, and practitioners have reported a positive impact of podcasts on their practices [60]. Creating a podcast is relatively inexpensive, with the minimum required piece of equipment being a dedicated microphone [61].

### Internet-Based Twitter Journal Clubs

Hosting monthly 24-hour Twitter discussions of recent publications demonstrate positive growth metrics over the course of the intervention in terms of active participation (number of Twitter profiles involved in the discussion), tweet volume, engagement, and impressions and has the potential to increase traffic to the journal’s website [59]. Unlike other Twitter strategies, internet-based journal clubs are based on conversations and real-time discussions [42]. In addition to discussing the study, internet-based journal clubs build relationships between the journal and the audience [49]. Chai et al [42] questioned whether participation in internet-based journal clubs improves knowledge of the topic and whether lessons learned are applied in real-world settings. Another caveat of internet-based journal clubs is that data are only captured on hashtags. As a consequence, important discussion points in tweets without the dedicated journal club hashtags may have been missed [48]. Successful implementation of a Twitter journal club also requires significant preparation and effort. For example, the development of a working group to select topics or manuscripts for tweet chats and having designated moderators to coordinate tweet chats and promote them [41]. If journals choose to implement the internet-based journal clubs strategy, recommendations to increase the likelihood of successful implementation thereof are ensuring that the authors of the studies participate in the web-based discussion [52], allowing opportunities for participants to provide feedback after the internet-based journal clubs [44], and choosing an optimal time to schedule the chats to allow participants from different time zones to join [52].

### Limitations and Future Research

The research designs of studies that tested the effectiveness of the same strategy differed. This made it difficult to draw definitive conclusions regarding Twitter strategy effectiveness [58]. Intervention periods for experimental studies were generally short (7-60 days), and it is unclear whether longer interventions will have greater impacts. In addition, although studies aimed at measuring the impact of different Twitter strategies on outcome measures, some studies also active or promoted articles on other social media platforms at the same time, thereby confounding the results. In terms of the systematic review itself, we tried to focus the search to Twitter studies only. As such, our Boolean terms were “social media” AND “Twitter” as opposed to “social media” OR “Twitter.” Admittedly, this may have excluded potential studies and may be considered a caveat of this review. Another potential limitation of our search strategy was the exclusion of the word *tweet* in the search terms.

### Conclusions

Twitter is a valuable science communication and marketing tool for academic journals to increase web-based visibility, promote research, and translate science to lay and scientific audiences. Four key Twitter strategies are implemented by medical journals: tweeting the title and link of the article, infographics, podcasts, and hosting monthly internet-based journal clubs. In this review, each strategy was successful in promoting publications but used different metrics to measure success. Thus, it is difficult to conclude which strategy is most effective. In addition, the four strategies have different costs and effects on dissemination and readership. We recommend that journals and researchers incorporate a combination of Twitter strategies to maximize research impact and capture audiences with a variety of learning methods.

## Appendix

Multimedia Appendix 1 Characteristics of included studies.

Multimedia Appendix 2 Quality of included studies using the Medical Education Quality Instruments.

## Abbreviations

PRISMA: Preferred Reporting Items for Systematic Reviews and Meta-analyses
