# Supplementary material for: The Use of Twitter by Medical Journals: Systematic Review of the Literature
Source: J Med Internet Res. 2021 Jul 28;23(7):e26378. doi: 10.2196/26378 (PMC8367184; doi:10.2196/26378)
Supplement: Multimedia Appendix 1 [file jmir_v23i7e26378_app1.docx]

| Study | Study design | Discipline | Aim | Participant sample and size | Primary outcomes | Duration | Methods | Primary findings | Social media strategy | Mersqi score |
| --- | --- | --- | --- | --- | --- | --- | --- | --- | --- | --- |
| Alotaibi et al (2016) [17] | Cross-sectional | Neurosurgery | To investigate the relationship between social media metrics and citation-based metrics of neurosurgical programs and journals | 38 journals | - SJR^a^ - H-index - Citation counts - Twitter followers - Facebook likes - Tweets - Twitter likes | Cross-sectional analyses | - Correlation analyses | - 11 (28.9%) journals had social media accounts. - Journals with social media accounts had significantly higher values of h-index and SJR (P<.001). - There was a significant correlation between SJR and tweets (r=0.614; P<.05) but not with Twitter followers, Twitter likes, or Klout score. - No significant correlation between h-index and Twitter followers, Twitter likes, tweets, or Klout score. | Observational | 9.5 |
| Amath et al (2017) [18] | Cross-sectional | Medical education | To explore the relationship between Altmetrics, access scores, and citations | 236 articles published in 2012 and 246 articles published in 2013 | - Citation - Altmetrics - Access counts (HTML views and PDF downloads) | All data were collected in September 2015. | - Correlation analyses | - Altmetric scores and Twitter scores were strongly correlated (r=0.96; P<.05). - Citation counts significantly correlated with access counts (0.62-0.77) and Mendeley downloads (0.61-0.81) in 2012 and 2013. - Citations were significantly correlated tweets(r=0.47), potential exposure to Twitter followers (r=0.42) and Altmetric score (r=0.57) in 2012 but not 2013. - Access counts were significantly correlated with Mendeley downloads (r=0.65-0.79), tweets (0.27-0.55), and Altmetric scores (0.30-0.52) but not with potential exposure to Twitter followers in 2012 and 2013. - Correlations were stronger in 2012 than in 2013 for each metric. | Observational | 9.5 |
| Chai et al (2018) [38] | Descriptive | Toxicology | To describe the development and experience of #firesidetox tweetchat as a virtual journal club to discuss and disseminate medical toxicology topics | Five tweet chats | - Twitter Impressions - Participant count - Tweets per tweetchat | 12 months | - A quarterly 1-hour tweet chat was developed featuring journal manuscripts. - Tweets containing the hashtag #firesidetox on the day of the session were analyzed. - Outcomes were downloaded 48 hours post tweet chat. | - Each tweet chat attracted a mean of 23.4 unique participants and generated a mean of 150 tweets and 329,200 impressions. - Impressions grew 300% from the first to fifth tweetchat. - 99 participants identified as pharmacists or toxicologists, 4 participants were residents, 4 were medical students, and 7 were members of the public. - 111 participants were from the United States, and 6 were international participants. - Medical education was the most popular theme of discussion. | Virtual journal club | 8 |
| Chang et al (2019) [19] | Cross-sectional | Pediatric surgery | To analyze the relationship between Altmetric score, citation counts, and impact factor | The top 10 most-cited articles from 14 pediatric surgery journals for the year 2012 (n=140) | - Citation counts - Journal impact factor - Altmetric score | All data were collected in 2015 | - Correlation analysis | - 13 out of 14 (93%) journals had a Twitter account. - Twitter was the primary source for Altmetric data. - Altmetric scores significantly correlate with citations in 2012 (r=0.189) and 2015 (r=0.238). - Altmetric scores do not significantly correlate with citations in 2012 but do in 2015 (r=0.2027) - Increasing age of a journals Twitter account increased the correlation between Altmetric score and citation counts in 2012 (r=0.299) and 2013 (r=0.512). | Observational | 10.5 |
| Chapman et al (2019)) [47] | Three-arm RCT^b^ with two arms crossed over | Surgery | To assess the role of plain English abstracts, disseminated via social media, in engaging patients and clinicians in the community of surgical research | 41 articles | - 14-day web-based engagement by the public - 14-day web-based engagement by health care professionals - Tweet impressions - Twitter detail expands - Twitter link clicks | August-December 2018 | - Articles were randomly allocated to one of three groups: English abstracts, visual abstracts, and standard tweets (1:1:1). - Abstracts were disseminated from the journal's Twitter account. - Abstracts were first tweeted according to their randomized allocation; plain English abstracts and visual abstracts were then crossed over and tweeted again after 14 days. - Text-only tweets were also tweeted again after 14 days to maintain continuity in outcome assessments. | - Visual abstracts received significantly more total engagements (P=.001), and engagements from health care professionals (P<.001) than plain English abstracts. - Visual abstracts did not receive significantly more engagement from the public than English abstracts (P=.082). - Visual abstracts received significantly more impressions (P=.001) and detail expands (P=.026) than plain English abstracts. - Standard tweets attracted more link clicks than plain English abstracts (P=.001) but no difference for visual abstracts (P=.148). - There was borderline significance toward visual abstracts attracting more link clicks than plain English abstracts. | Visual abstracts | 10.5 |
| Cosco (2015) [14] | Cross-sectional | General medical journals | To examine the relationship between scientific merit (journal impact factor) and Twitter celebrity (number of followers) in medical journals | 153 general medical journals | - Impact factor - Citation counts - Twitter presence | Cross-sectional | - Correlation analyses - Regression analyses | - 28% of journals had Twitter profiles - Significant correlations between number of followers and journal impact factor (r=0.68; P<.001) and journal citations (r=0.69; P<.001). | Observational | 9.5 |
| Dardas et al (2019) [20] | Cross-sectional | Nursing | To analyze the association between articles’ Altmetric score, publication characteristics, citation counts, and publishing journal metrics | The top 100 articles by Altmetric score (n=100) | - Altmetric scores - Citation count | Cross-sectional | - Thematic analysis | - Articles were mostly discussed on Twitter. - Significant relationship between articles Altmetric attention score and citation count on Scopus (r=0.342; P=.001) and Web of Science (r=0.369; P=.001). | Observational | 9.5 |
| Eysenbach (2011) [21] | Prospective longitudinal | Medical internet research | - To explore the content and characteristics of tweets discussing or mentioning research articles and their timing relative to the publication date of an article - Identify suitable metrics to describe propagation of new evidence through social media networks and - To explore how the proposed metrics correlate with traditional citations | 4208 tweets and 286 JMIR articles | - Twitter mentions - Citation counts | All tweets linking to journal articles sent between July 2008 and November 2011 by JMIR were mined. Citation counts were harvested in November 2011, 17-29 months after the cited papers were published. | - Tweet distribution analysis - Tweetation-citation correlation analysis. | - The Pearson correlation between tweetation and Google Scholar citations was moderate and statistically significant (r=0.42 to 0.72), but less clear for Scopus and rank correlations. - Highly tweeted articles are 11 times more likely to be highly cited than less tweeted articles. | Observational | 8.5 |
| Fox et al (2015) [ 48] | 2-arm RCT | Cardiology | To assess whether social media exposure improves article impact metrics | 130 original research papers | - 30-day pageviews | 30 days | - Articles were randomized to either social media exposure or no social media exposure (control). - Social media exposure consisted of Facebook and Twitter posts through official circulation social media accounts, Monday through Thursday weekly. - Posts were written in English to convey the main point of each article with a link to the full-text version of the article, and when possible, a key figure was included. | - No significant difference was observed in the median number of 30-day page views between the intervention arm (499.5) versus the control arm (450.5; P=.38). | Basic article promotion | 10.5 |
| Fox et al (2016) [49] | Two-arm RCT | Cardiology | To determine whether a higher intensity social media strategy could increase the number of times an original article was viewed | Original research articles published in circulation (n=152) | - 30-day pageviews | January 13, 2015, to September 26, 2015 | - Original articles were randomized with 1:1 allocation to either social media or no social media group (control). - Social media exposure consisted of Facebook and Twitter posts on the journal's account. - Posts were written in clear English, included a key figure or image if possible, and appeared on the web Monday through Thursday, contemporaneous with the publication of the articles. - To increase social media intensity, a larger base of followers built, and posts were presented in triplicate and boosted on Facebook, and retweeted on Twitter. | - Median 30-day pageviews were not significantly different between social media and control groups (P=.38). | Enhanced article promotion | 10.5 |
| Gardhouse et al (2017) [39] | Descriptive | Geriatric medicine | To describe the first year of the #garimedjc, a monthly Twitter-based complement to the traditional-formal geriatric medicine journal club | 12 journal club sessions | - Participants - Tweets - Twitter Retweets - Twitter Replies - Twitter impressions - Origin and occupation of participants and followers | - 12 months | - A monthly 1-hour live, 23-hour asynchronous Twitter-based journal club was hosted. - Tweets containing the hashtag #gerimedjc on the day of the session were analyzed. - 2 articles were discussed per session. - Outcomes were collected immediately (24 hours) after each session. | - @gerimedjc grew from 80 followers in month 1 to 541 followers in month 12. - Followers represented 6 continents and were occupationally diverse. - Tweet volume grew from 23 tweets in the first session to 121 tweets in 12th session (median 127). - Impressions grew from 10,486 in session 1 to 74,259 in session 12 (median 64,560). - Active participation grew from 5 participants in the first session to 25 participants in the 12th session (median 21). - Participants comprised physicians (45%), trainees (17%), organizations (16%), and allied health members (9%). | Virtual journal club | 9 |
| Hawkins et al (2014) [63] | Descriptive | Radiology | To describe the journal’s social media initiative, evaluate Twitter user metrics associated with these tweet chats, and evaluate temporally related journal website activity | Six tweet chat sessions | - Participants - Tweets - Twitter Impressions - Twitter engagements - Twitter retweets - Percentage of tweets with weblinks | 24 months | - 1-hour monthly journal clubs were hosted on Twitter by @jacr. - Tweets containing #jacr were analyzed. | - Each session had a mean of 33 participants; generated a mean of 444 tweets and a mean of 1,163712 impressions. - On average, 45.4% of participants were non–radiology-related, 18 out of 33 were radiology related, and 6 out of 18 were trainees. - Monthly journal website article views increased 31.4%, journal website visits increased 25.5%, and unique visits increased 20%. - Mean monthly journal’s website visits and pageviews per month increased 321% and 318%, respectively. | Tweet chat | 8 |
| Hawkins et al (2017) [40] | Three-arm RCT | Radiology | To evaluate the impact of increasing levels of social media engagement on page visits and weblink clicks for content published in the J*ournal of the American College of Radiology* | 428 articles | - 7 and 30-day pageviews visits - 7 and 30-day weblink clicks | 30 days | - Articles were randomized to one of three intervention arms: the basic Twitter arm, the enhanced Twitter arm, and the control arm. - The basic Twitter arm consisted of a single tweet from @jacrjournal Twitter account per day. - In the enhanced Twitter arm, articles were assigned to one of four teams. Each team was assigned 1 article each day, and each member was instructed to post about it (four posts per article). - No social media activity was planned for the control group. | - Weekly page visits were significantly greater for the enhanced Twitter group than the basic Twitter group and control (P<.001). - The number of monthly page visits received by the enhanced Twitter group was significantly greater than the basic Twitter group (P<.02), but it was not significantly different from the control group (P>.05). - The number of link clicks was significantly greater for the enhanced Twitter arm than the basic Twitter arm after 7 days (+14.7 clicks) and 30 days (+15.7 clicks). | Enhanced article promotion | 10.5 |
| Hayon et al (2019) [22] | Cross-sectional | Urology | To quantify the relationship between the number of Twitter mentions and the number of academic citations | 213 original research articles from 7 urologic journals published in January 2015 | - Citations - Twitter mentions | - Data collection occurred in February 2018 | - ANOVA - Bivariate fit analysis | - Publications with at least one Twitter mention were significantly associated with increased Scopus (two-fold increase P<.01) and Google Scholar (2.3-fold increase P<.01) citations. | Observational | 9.5 |
| Huang et al (2018) [50] | Two-arm RCT | Emergency medicine | Determine the effect of a social media strategy using infographics on the readership and dissemination of research articles of a medical journal | 24 original articles published in the *Canadian Journal of Emergency Medicine* (CJEM) | - Altmetric score - Abstract Pageviews - Full-text pageviews | July 2016 to June 2017 | - Four original articles were chosen from each issue of CJEM. Articles were randomized to infographic or control group. Each article was promoted within 1-2 weeks after its official publication in a CJEM issue - Infographics were published via (1) a tweet from both the CJEM and Canadiem Twitter accounts; (2) post on both the CJEM and Canadiem Facebook pages; and (3) blogpost on the Canadiem website - Control articles were posted from CJEM Twitter and Facebook accounts with a picture of the article's abstract. | - The infographic group had a significantly larger change in Altmetric score (mean 26 vs 3; P<.0001) and abstract views (379 vs 176; P<.001) than the control group. - There was no statistically significant difference in full-text views between groups (mean 50 for infographic vs 25 for control; P=.36). | Infographics or visual abstracts | 10.5 |
| Hughes et al (2017) [23] | Cross-sectional | Orthopedic surgery | To analyze the prevalence and activity of trauma and orthopedic surgery journals on Twitter | The top 50 per 2016 if trauma and orthopedic journals | - Impact factor - 1-year Twitter activity | - Cross-sectional - Data collection occurred in 2017 | - Student t tests - Contingency table - Correlation analysis | - 44% of journals had a dedicated Twitter profile. - The mean Impact Factor (P=.007) and mean Altmetric score (P<.001) was higher for journals with dedicated Twitter profiles than those without (P=.007). - There was no correlation between the age of a journals Twitter account and their Impact Factor. - Twitter Klout score is significantly correlated with Impact Factor (r=0.3257; P=.016) - Twitter followers is significantly correlated with Klout score (r=0.339; P=.061) but not Impact Factor (r^2^=0.339; P=.061) - The number of years a journal had been on Twitter had no association with Twitter Klout score. - The number of tweets a journal produced on Twitter per day had no correlation with its impact factor or Twitter Klout score. - The number of retweets was not associated with if but was associated with having a higher Klout score (r=0.509; P=.008). - Journal Altmetric score is significantly correlated with Impact Factor in 2015 (r^2^=0.334; P=.009) and 2016 (r^2^=0.310; P=.015) - Journals with higher numbers of tweets and a higher number of retweets (r^2^=0.463; P=.015) were also associated with higher Altmetric scores. | Observational | 9.5 |
| Ibrahim et al (2017) [51] | A prospective, case-controlled cross over study | Surgery | To compare tweets that included only a title of the article versus tweets that contain the title and a visual abstract | 44 original articles | - Impressions - Twitter retweets - Article visits | July 2016 to December 2016 | - 44 original research articles were tweeted from the Twitter account of a medical journal in two formats: as the title of the article only and as the title with a visual abstract - Half of the articles were tweeted as title alone, then after a 4-week ‘‘washout’’ period, the same article was also tweeted as a visual abstract. The other half of the articles were tweeted on the same protocol, but in the opposite order. | - The mean number of impressions received was 7.7-fold greater for the visual abstract group compared with articles in the title-only group(P<.001). - The mean number of retweets was 8.4-fold greater for the visual abstract group versus the control group (P<.001). - The mean number of article visits was 2.7-fold greater for the visual abstract group compared with the control group (65.6; P<.001). - The mean number of impressions for nonvisual abstract tweets after the visual abstract strategy was implemented increased from 2417 to 4574.9 (P<.001). - The annals Twitter account acquired 3455 new followers throughout during the intervention period. | Visual abstracts or infographics | 10.5 |
| Jeong et al (2019) [24] | Cross-sectional | Coloproctology | To investigate the association between Twitter exposure and the number of citations for coloproctology articles | All original articles published between June 2015 to May 2016 in three coloproctology journals (n=404) | - Impact factor - Citation of article | Cross-sectional analyses | - Univariate analyses - Multivariate analyses | - 50% of the journals actively used Twitter. - Citation rates were significantly higher for articles exposed on Twitter versus those not exposed on Twitter (P<.001). - Review articles were cited significantly more than other study designs (P<.001). - The mean number of authors per article was significantly greater for articles cited more than 5-times than those cited five times or less (P=.008). - Twitter exposure (P<.001), type of journal (P=.002), and study design (P=.001) were associated with high or low levels of citation (univariate analyses). | Observational | 9.5 |
| Kelly et al (2016) [25] | Retrospective cross-sectional | Radiology | To analyze the use of Twitter by leading radiology journals | The top 50 journals per 2014 impact factor (n=50) | - Tweets - Twitter retweets - Twitter followers - Age of the Twitter profile - Klout score - Impact factor | - Cross-sectional analyses - Data collection took place in 2016 | - Correlation analyses | - 28% of journals had dedicated Twitter profiles, 50% of journals were associated with societies that had profiles, and 22% had no presence on Twitter. - Journals with a presence on Twitter had higher if than those without profiles (3.37 vs 2.14; P<.001). - There was no significant difference between Impact Factor of journals with dedicated Twitter accounts and those with affiliated societies (P=.47). - Journals with links on their website to their social media platforms were associated with high if (P=.2). - A larger number of Twitter followers is associated with a higher impact factor (r=0.581; P=.029). - Klout score and if was not significantly correlated (P=.244). - The age of a Twitter profile was not associated with impact factor (P=.23) or Klout score (P=.42). - Number of tweets per year was not associated with the impact factor (P=.56). | Observational | 9.5 |
| Koo et al (2019) [41] | Cross-sectional analysis | Urology | To assess the impact of visual abstracts on the dissemination of and reader engagement with articles on social media from a medical journal | 1445 tweets | - Twitter Impressions - Twitter engagement - Twitter engagement rate | Cross-sectional | - Tweets originating from a medical journal between a 2-year period were reviewed. - Tweets were coded in the following categories: containing a (1) visual abstract, (2) nonvisual abstract graphic, (3) no media, and (4) were about nonresearch articles - Impact metrics were compared 4 months before and after the introduction of visual abstracts | - Visual abstract tweets received significantly greater impressions (3107 vs 2741 vs 2289), engagements (128 vs 113 vs 58) and engagement rate (3.8% vs 3.6% vs 2.3%) nonvisual abstract tweets (P<.001) and no media tweets (P<.001) - Compared with tweets without media, visual abstract tweets increased readers’ overall engagement rate by 65% - Visual abstract had a positive spill over effect on research articles: after visual abstracts were introduced, nonvisual abstracts tweets received a 31% increase in impressions, a 152% increase in engagements, and a 62% increase in full article visits. | Visual abstracts/infographics | 8.5 |
| Luc et al (2019) [42] | Descriptive | Thoracic surgery | To describe the experience of a virtual journal club on Twitter | 13 tweet chats | - Twitter analytics - Hashtag analytics | 13 months | - 1-hour live tweet chats addressing interesting topics with relevant papers from both journals. - Tweets containing #tssmn were analyzed. | - Hashtag #tssmn has a total of 17,181 total tweets, 2100 users, and 32,226,280 impressions, with peaks in tweeting activity of #tssmn corresponding to tweet chats. - Thirteen 1-hour tweet chats drew a total of 489 participants, 5195 tweets, 17,297,709 total impressions. - Tweet chat participants identified as doctors (47%), organization advocate/support (11%), patients (3%), and unknown (10%). - Tweet chats with the lowest impression were help at 5 PM as compared with later times in the day. | Tweet chat | 8 |
| Luc et al (2020) [52] | Two-arm RCT | Thoracic surgery | To examine the effect of scheduled tweeting via tssmn on nontraditional bibliometrics of dissemination | 112 original scientific articles from two cardiothoracic surgery journals were published between 2017-2018 | - 14-day Altmetric score change - 7-day change in Twitter analytics | 14 days | - Articles were randomized (1:1) to the either tweeted or nontweeted (control) group. - Four articles were tweeted per day by a tssmn delegate and retweeted by other delegates (n=11) and @tssm for 14 days. - The headline and social media summary were standardized in simple English to convey the authors and institutions involved in the study, the main point of each article, and a link to the full-text version on the respective journal's website. - No social media activity were planned for the control. | - Tweeted articles received higher tweets on day 7 (P<.001) resulting in improved Altmetric score (P<.001) and Mendeley reads (P<.001). - Posttweet Altmetric scores (P<.001), Mendeley reads (P<.001), and number of tweets (P<.001) were significantly greater than pretweet, for tweeted articles. - The reach of tweeted articles not only physicians in the social media community (P<.001) but also to members of the public (P<.001). - Tweets with a photo received more media views (P<.001) and media engagements (P<.001) and trended toward increased link clicks (P=.08). - Tweeting at 1 PM generated the highest and 9 PM the lowest reach to both physicians (P=.004) and members of the public (P=.022), respectively. | Basic article promotion | 10.5 |
| Mcginnigle et al (2017) [43] | Descriptive | General internal medicine | To analyze activity and impact of the general internal journal club #genmedjc (@gimjclub) | Twitter-based general internal medicine journal club | - Number of total and unique users and number of tweets, impressions and engagement. | 12 months | - A 1-hour monthly journal club was hosted on Twitter. - Tweets containing the hashtag #genmedjc on the day of the session were analyzed. - 1 article was discussed per session. | - A total of 1543 tweets were sent across the sessions, averaging 128 tweets per session. - Each session had roughly 200-400 impressions, 6-12 engagements, and 2-14 users of which, roughly, 7 were new users. - The journal club's Twitter profile followers increased from 880-5000 during the 12 months. - Participants represented over 37 countries. | Twitter-based journal club | 8 |
| Mullins et al (2020) [26] | Cross-sectional analysis | General surgery | To evaluate the relationship between Altmetric scores, journal impact factor, and citation count | The top 10 most-cited articles from 12 journals in 2013 and 2016 (n=120) | - Citation counts - Journal impact factor - Altmetric score | All data were collected in 2018 | - Correlation analysis | - Altmetric score is significantly correlated with citation number in 2013 (r=0.462) but not 2016. - Altmetric score was significantly correlated with journal impact factor in 2013 (r=0.439) and 2016 (r=0.425). - Older Twitter accounts were not significantly associated with increasing correlation between Altmetrics score and citation in both years (P>.05). - Twitter mentions were the primary source for Almetric data. | Observational | 10.5 |
| Munoz-velandia et al (2019) [27] | Cross-sectional | Endocrinology | To assess the correlation between SJR and metrics of presence on social networks | 232 endocrinology journals | - SJR - Number of followers - Number of tweets - Age of social media account | All data collection occurred in 2019 | - Correlation analyses | - 28 journals had social networks (12.1%), and 100% of these journals had a Twitter account. - The median SJR (1.53 vs 0.60; P<.01), and h-index (58.5 vs 22; P<.01) were significantly higher among journals with social networks. - Significant correlation between the number of followers on Twitter and the SJR (r=0.60; P<.05), and it was better in journals with more than 500 publications in the last 3 years (r=0.85; P<.05). - The number of followers/year was moderately correlated with SJR (r=0.57; P<.05) and strongly correlated with journals with >500 publications in the last 3 years (r=0.85; P<.05). - The number of tweets also had a moderate correlation with the SJR (r=0.59; P<.05). | Observational | 9.5 |
| Nason et al (2015) [28] | Cross-sectional | Urology | To assess the use of Twitter by urological journals | 33 urological journals | - 2012 if - Twitter metrics - Klout score | - Cross-sectional analyses - Data collection took place april 2014 | - Correlation analyses | - A Twitter profile was associated with a higher mean impact factor (P<.013). - There was no correlation between the number of followers with impact factor (P=.534) or with the number of tweets (P=.647) or with the age of the Twitter profile (P=.433). | Observational | 9.5 |
| Ni hlci et al (2020) [44] | Descriptive | Thoracic surgery | To characterize the experience of the trainee tssmn group tweet chat–based journal club | Three tweet chats | - Number of tweets, participants, most popular tweets, and impressions for each tweet chat | - December 2017-May 2018 | - Each tweet chat discussed 2-4 publications. - Tweets containing the hashtag #tssmn on the day of the session were analyzed. - Outcomes were downloaded from symplur. | - Each tweet chat had a mean of 40 participants, generating a mean of 497 tweets with a mean of 809,746 impressions. - Tweet chat participants consisted of doctors (40%), advocate organizations (17%), unknown (10%), caregiver/advocate (5%), other health care individual (5%), health care organizations (5%), media organizations (5%), patients (2%), researchers (2%), media (5%), and other (2%). | Virtual journal club | 8 |
| Nocera et al (2019) [29] | Cross-sectional analysis | Urology | To analyze the relationship between Altmetric scores, journal impact factor, and citation counts | Top 10 most-cited articles from the top 15 if (n=300) in 2013 and 2016 | - Citation counts - Journal impact factor - Altmetric score | All data were collected in 2019 | - Correlation analysis | - 10 out of 15 (66%) journals had established Twitter accounts. - Altmetric scores significantly correlated with article citation counts (r=0.164) but not with journal impact factor in 2013. - Altmetric scores were significantly correlated with article citation number (r=0.268) and journal impact factor (r=0.201) in 2016. - Longer standing Twitter accounts were associated with increasing correlations between Altmetrics score and bibliometrics. - Twitter was the most used medium in which articles were shared in 2013 and 2016. | Observational | 10.5 |
| O’Kelly et al (2017) [30] | Cross-sectional | Pediatric urology | Assess the impact of social media platforms on the impact factor of pediatric urology journals | 50 urological journals and 39 pediatric journals | - 4-year Impact Factor (2012-2014) - Presence of Twitter account | - Cross-sectional analyses - Data collection took place in 2016 | - Correlation analyses - Regression analyses | - Journals offering more than three social media outlets had significantly higher Impact Factor (P=.017). - The presence of a Twitter account between 2014 and 2015 was associated with an increase in impact factor compared with a decrease in Impact Factor in the absence of a Twitter account (P=.022). | Observational | 9.5 |
| Ordonez-gutierrez et al (2020) [31] | Cross-sectional analysis | Immunology | To analyze the relationship between the traditional impact factor and activity on social media | 156 journals | - SJR - H-index - Altmetric score | All data were collected in 2019 | - Correlation analysis | - SJR was significantly correlated with the number of tweets (r=0.63) and number of followers (r=0.83). | Observational | 9.5 |
| Ortega (2017) [12] | Cross-sectional | Science journals | To analyze the relationship between the dissemination of research papers on Twitter and its influence in research impact | 4176 articles published in 2013 from 350 journals (n=4176) | - Type of Twitter account - Article tweets - Article citations | - Cross-sectional analyses - Data collection took place in 2016 | - Student t test for independent samples - Regression analyses | - Items from journals with an own account are more tweeted than papers from other journals (P<.001). - Articles from the journal (89%) and the owner account (88%) are more cited, whereas articles from journals not registered on Twitter are less cited (52%; P<.001). - Journals with their own account could generate 5.5% more tweets and 3.4% more citations by increasing followers by 10%. | Observational | 9.5 |
| Patino-hernandez et al (2019) [31] | Cross-sectional | Pulmonary | To describe the correlation between SJR impact factor and use of social media by journals | 140 pulmonary medicine journals | - SJR - H-index - Social networks (Twitter, Facebook, Instagram, Youtube) | - Cross-sectional analyses - Data collection took place in February 2019 | - Correlation analyses | - The H-index is significantly higher in journals with social network account (P<.01). - SJR was not significantly different between journals with or without a social network account (P=.279). - Twitter was the most frequently used social network (22.8%). - Number of Twitter followers were significantly correlated with SJR (r=0.46; P<.05). - Number of tweets was not significantly correlated with SJR but was significantly correlated for open access journals (r=0.90; P<.05). - SJR and followers/year was significantly correlated (r=0.463; P<.05) and this correlation improved for nonopen access journals (r=0.483; P<.05). | Observational | 9.5 |
| Rosenkrantz et al (2017) [33] | Cross-sectional | Radiology | Compare citation counts and alternative Altmetric score for radiology articles | 892 articles from four journals published in 2013 | - Citation count - Altmetric scores | All data were collected in 2016. | - Correlation analysis | - Weak correlation between citation count with Altmetric attention Scores (r=0.2) and the number of Twitter mentions (r=0.17). - 34.2% of articles had at least one Twitter mention. | Observational | 9.5 |
| Smith et al (2019) [34] | Cross-sectional | Gastroenterology | To analyze the relationship between social media activity and number of citations | Original research articles published in gie from 2010 to 2016 (n=2631) | - Number of citations - Altmetric score - Social media exposure (Facebook, Twitter, and LinkedIn) | Data were analyzed on February 28, 2017. | - Regression analysis | - Twitter was the most used social media platform. - No significant difference in Altmetric attention score between articles that were cited and those that were not (P=.239). - The most profound predictor of article citation was whether an article was tweeted (OR^d^ 14.16, 95% CI 8.93-22.45; P<.001). - No significant association was observed between the number of tweeters and whether an article was cited. - The number of tweeters (β=2.3; P=.22) was significantly associated with the citation rate of published articles. | Observational | 8.5 |
| Thangasamy et al (2014) [45] | Descriptive | Urology | To describe the 12-month experience of #urojc virtual journal club | 12 journal club sessions | - Number of total and unique users - Number of tweets - Qualitative analysis of the relevance of tweets | 12 months | - A 48-hour monthly journal club was hosted on Twitter and moderated by Twitter account @iurojc. - Tweets containing the hashtag #iurojc on the day of the session were analyzed. - 1 recently published article was discussed in each session. - Outcomes were compiled directly after each session. | - A total of 189 unique users representing 19 countries and 6 continents participated in #urojc. - Each session had a mean of 39 monthly participants from a mean of 7 countries and 3 continents. - There was a mean of 14 new participants each month. - Each session generated a mean number of 195 tweets which lead to a mean of 130,832 impressions. - The @iurojc moderator account has accumulated >1000 followers after 12 months. | Twitter-based journal club | 8 |
| Thoma et al (2018) [53] | Three-arm RCT | Emergency medicine | Assess the effect that promoting articles using podcasts and infographics has on the dissemination and readership of CJEM articles | 29 original articles | - Altmetric score - Abstract views - Full-text page views - Numbers needed to view. | — | - Publications in the 2015 and 2016 issues from “original research” and “state of the art” sections were selected for podcast and infographic promotion based on perceived interest to emergency physicians. - A control group was composed retrospectively of articles from the 2015 and 2016 issues with the highest Altmetric score that received standard Facebook and Twitter promotions. | - Abstract views significantly increased for podcasts (mean 1795; 95% CI 1135-2455) and infographic strategies (mean 590; 95% CI 361-819) in comparison with the control (mean 257; 95% CI 159-354) - Podcasts (mean 61; 95% CI 42-80) and infographics (mean 32; 95% CI 19-43) strategies significantly increased article Altmetric score when compared with the control (mean 12; 95% CI 8-15). - The mean number of full-text views was not significantly different between control (mean 73; 95% CI 38-109), infographic (mean 65; 95% CI 33-98) and podcast group (mean 431; 95% CI 0-1031). | Podcasts and infographics | 9.5 |
| Tonia et al (2016) [54] | Two-arm RCT | Public health | To investigate whether exposing scientific papers to social media has an effect on article downloads and citations | 133 original articles published in *International Journal of Public Health* between December 2012 and December 2014 | - The number of full article downloads - The number of article citations | 2 years | - Articles were randomized to social media exposure or control group. - Each intervention article received exposure from three different venues (blog, Twitter, and Facebook) and applied at 3 different time points: 2 weeks after the first intervention and 10 weeks after the second intervention. - Posts were written in plain language, highlighted the main point of the paper, and provided a link to it. Authors of the paper were tagged if they had a Twitter account. | - The mean number of downloads did not significantly differ between the social media group (191.8), and no social media group (202.7) did not differ significantly (p=0.60). - The mean number of citations for the social media group (0.85) and no social media group (0.77) were not significantly different (P=.88). - The number of downloads significantly correlated with the number of citations for all papers (P=.529; P<.001) both in the social media and control group, and this correlation was stronger in the social media group (P=.67; P<.001). | Basic article promotion | 10.5 |
| Truegar and Bokarius (2018) [11] | Observational before-and-after-analysis | Emergency medicine | To compare the change in web-based views before and after the intervention | 267 articles were posted in the preintervention period, and 197 posted in the postintervention period | - Pageviews per article from Twitter each month - Total page views per month | The preintervention period spanned January 1, 2013, to June 30, 2014, and the postintervention period spanned July 1, 2014, to July 31, 2015 | - The intervention consisted of a nine-person social media team actively posting links to articles in press using their social media accounts. | - The mean number of web page views received from Twitter increased 273% after the intervention (10,522 vs 22,971). - The number of page views from Twitter per article increased 294% (33 vs to 130) (P<.001). - There was no detectable difference in total page views (116,114 vs 132,223). | Basic article promotion | 9 |
| Wadhwa et al (2017) [36] | Retrospective cross-sectional | Neuroradiology | To identify the characteristics of A*merican Journal of Neuroradiology’s* tweets associated higher user engagement rates | 1032 tweets posted from @theajnr | - Twitter impressions - Twitter engagements - Twitter Engagement rate | August 2015-July 2016 | - Tweets were characterized by month, time of day, weekend versus weekday, presence or absence of an embedded image or hashtag, and type of tweets. - Statistical analysis included a multivariate logistic regression model. | - The presence of an image increases the tweet engagement rate by 28.75 times (P<.001). - The presence of a hashtag increased the rate by 3.27 times (P<.001). - Tweets published in the morning were twice as likely to have a high engagement rate compared with those published in the afternoon hours (P=.001). - There was no significant difference in tweets posted on the weekend versus a weekday (P=.91), evening compared with afternoon (P=.445), night compared with the evening (P=.254), or according to the month. - The “case” tweet type was 140-fold more engaging than other tweet types (P<.001). | Basic article promotion | 8.5 |
| Wang et al (2017) [45] | Cross-sectional | Neurosurgery | Examining the relationship between social media metrics and Altmetric scores of various neurosurgical journals | Original articles published in 16 neurosurgery journals between 2010 and 2017 | - Article Altmetric score - Journal social media presence - Number of tweets | - Cross-sectional analyses | - Correlation analyses | - Journals with social media accounts (Facebook and Twitter) had significantly higher Altmetric scores for their articles compared with those without (P<.001). - Out of all variables for web-based mentions, the number of tweets had the strongest correlation with Altmetric scores (r=.227; P<.01). | Observational | 9.5 |
| Widmer et al (2019) [55] | Two-arm RCT | General internal medicine | To study the effect of a planned social media strategy on access of web-based articles of a medical journal | 68 articles published in *mayo clinic proceedings* | - 30-day and 60-day website visits and full-text article downloads | 60 days | - Articles randomized with 1:1 allocation to social media or no social media on a per month basis. - The social media promotion began on the date of each articles’ web-based publication. Each article had 7 social media posts throughout the entire month (2 Facebook, 4 Twitter, and 1 LinkedIn). - Each post was in English, included images when available, and contained links to full-text version.. | - Median total websites were significantly higher in some after 30-days (1070 vs 265; P<.001) and 60 days (194 vs 69.5; P=.009). - Median full-text downloads were significantly higher in some after 30 days (1042 vs 142; P<.001) and 60 days (115 vs 21; P<.003) - Median some-associated access to articles through the 60-day period for Twitter (90 vs 1; P<.001), Facebook (526 vs 2.5; P<.001) and LinkedIn (31.5 vs 0; P<.001) was higher for some articles. | Basic article promotion | 10.5 |
| Wong et al (2018) [36] | Cross-sectional | Otolaryngology | To evaluate the use of Twitter by the relationship between social media metrics and measures of academic impact | Top 50 journals per 2016 SJR | - Tweets - Klout Score - SJR - H-index | Cross-sectional analyses | - Correlation analyses | - 36% of journals had Twitter profiles. - Journals with Twitter accounts had significantly higher SJR (P=.03) and H-index (P=.01) scores compared with journals without. - Older Twitter accounts were associated with higher Klout scores (P=.04). - There is a significant correlation between a journal’s Klout score and their SJR rank (r=0.64; P=.004) but not with H-index (r=0.42; P>.05). - Number of Twitter followers is significantly correlated to H-index (r=0.6; P=.009) but not with SJR (r=0.36; P>.05). - The number of tweets sent by journals is significantly correlated with SJR (r=0.51; P=.03) and H-index (r=0.59; P=.01). | Observational | 9.5 |
| Zhou et al (2018) [37] | Cross-sectional | Plastic surgery | To analyze the relationship between social media use and the dissemination of research across nontraditional channels | Top 10 articles based off Altmetric attention score from 10 plastic surgery journals (n=100) | - Altmetric score - Article age - Journal impact factor | All data were collected in 2016. | - Correlation analyses - Regression analyses | - The most popular social media platform for journals is Twitter (7 out of 10). - The number of Twitter followers is strongly correlated to the number of tweets (r=0.84; P<.05) and likes (r=0.94; P<.05). - For each 1000 additional Twitter followers, the Altmetric score increases by a factor of 1.72 (95% CI 1.076-2.749). - No significant relationship between impact factor and Altmetric score. | Observational | 9.5 |

Cells that are left empty mean that the category was not applicable to that specific paper

^a^SJR = SCImago Journal Rank.

^b^RCT: randomized controlled trial.

^c^ANOVA: analysis of variance.

^d^OR: odds ratio.
