# Supplementary material for: The Use of Twitter by Medical Journals: Systematic Review of the Literature
Source: J Med Internet Res. 2021 Jul 28;23(7):e26378. doi: 10.2196/26378 (PMC8367184; doi:10.2196/26378)
Supplement: Multimedia Appendix 2 [file jmir_v23i7e26378_app2.docx]

**Medical Education Research Quality Instrument ‐ for** *quantitative studies*

| Domain | MERSQI Item | Score | Max Score |
| --- | --- | --- | --- |
| 1. Study design | Single group cross‐sectional or single group posttest only | 1 | 3 |
|  | Single group pretest & posttest | 1.5 |  |
|  | Nonrandomized, 2 groups | 2 |  |
|  | Randomized controlled trial | 3 |  |
| 2. Sampling | *2.1) Institutions studied*: |  | 1.5 |
|  | 1 | 0.5 |  |
|  | 2 | 1 |  |
|  | 3 | 1.5 |  |
|  | *2.2) Response rate, %:* |  |  |
|  | Not applicable |  |  |
|  | <50 or not reported | N/A |  |
|  | 50‐74 | N/A |  |
|  | >75 | N/A |  |
| 3. Type of data | Assessment by participants | 1 | 3 |
|  | Objective measurement | 3 |  |
| 4. Validity of evaluation instrument | *4.1)Internal structure:* |  | N/A |
|  | Not applicable |  |  |
|  | Not reported | 0 |  |
|  | Reported | 1 |  |
|  | *4.2)Content:* |  |  |
|  | Not applicable |  |  |
|  | Not reported | 0 |  |
|  | Reported | 1 |  |
|  | *4.3)Relationships to other variables:* |  |  |
|  | Not applicable |  |  |
|  | Not reported | 0 |  |
|  | Reported | 1 |  |
| 5. Data analysis | *5.1)Appropriateness of analysis:* |  | 3 |
|  | Inappropriate for study design or type of data | 0 |  |
|  | Appropriate for study design, type of data | 1 |  |
|  | *5.2) Complexity of analysis:* |  |  |
|  | Descriptive analysis only | 1 |  |
|  | Beyond descriptive analysis | 2 |  |
| 6. Outcomes | Satisfaction, attitudes, perceptions, opinions, general facts | 1 | 3 |
|  | Knowledge, skills | 1.5 |  |
|  | Behaviors | 2 |  |
|  | Patient/health care outcome | 3 |  |
| Total possible score* |  |  | 13,5 |

*Scores range from 5 to 13.5

Appendix 1. Quality of included studies using the Medical Education Quality Instruments

|  | 1 | 2.1 | 2.2 | 3 | 4 | 5.1 | 5.2 | 6 | Total |
| --- | --- | --- | --- | --- | --- | --- | --- | --- | --- |
| Alotaibi, 2016 | 1 | 1,5 | N/A | 3 | N/A | 2 | 1 | 1 | 9,5 |
| Amath, 2017 | 2 | 0,5 | N/A | 3 | N/A | 2 | 1 | 1 | 9,5 |
| Chai, 2018 | 1,5 | 0,5 | N/A | 3 | N/A | 1 | 1 | 1 | 8 |
| Chang, 2019 | 2 | 1,5 | N/A | 3 | n/A | 2 | 1 | 1 | 10,5 |
| Chapman, 2016 | 3 | 0,5 | n/A | 3 | N/A | 2 | 1 | 1 | 10,5 |
| Cosco, 2015 | 1 | 1,5 | N/A | 3 | n/A | 2 | 1 | 1 | 9,5 |
| Dardas, 2019 | 1 | 1,5 | N/A | 3 | N/A | 2 | 1 | 1 | 9,5 |
| Eysenbach, 2011 | 1 | 0,5 | N/A | 3 | N/A | 2 | 1 | 1 | 8,5 |
| Fox, 2015 | 3 | 0,5 | n/A | 3 | N/A | 2 | 1 | 1 | 10,5 |
| Fox, 2016 | 3 | 0,5 | N/A | 3 | N/A | 2 | 1 | 1 | 10,5 |
| Gardhouse, 2017 | 1,5 | 0,5 | N/A | 3 | N/A | 2 | 1 | 1 | 9 |
| Hawkins, 2014 | 1,5 | 0,5 | N/A | 3 | N/A | 1 | 1 | 1 | 8 |
| Hawkins, 2017 | 3 | 0,5 | N/A | 3 | N/A | 2 | 1 | 1 | 10,5 |
| Hayon, 2019 | 1 | 1,5 | N/A | 3 | N/A | 2 | 1 | 1 | 9,5 |
| Huang, 2018 | 3 | 0,5 | N/A | 3 | N/A | 2 | 1 | 1 | 10,5 |
| Hughes, 2017 | 1 | 1,5 | N/A | 3 | N/A | 2 | 1 | 1 | 9,5 |
| Ibrahim et al, 2017 | 3 | 0,5 | N/A | 3 | N/A | 2 | 1 | 1 | 10,5 |
| Jeong, 2019 | 1 | 1,5 | N/A | 3 | N/A | 2 | 1 | 1 | 9,5 |
| Kelly, 2016 | 1 | 1,5 | N/A | 3 | N/A | 2 | 1 | 1 | 9,5 |
| Koo, 2019 | 1 | 0,5 | n/A | 3 | N/A | 2 | 1 | 1 | 8,5 |
| Luc, 2019 | 1,5 | 0,5 | N/A | 3 | N/A | 1 | 1 | 1 | 8 |
| Luc, 2020 | 3 | 0,5 | N/A | 3 | N/A | 2 | 1 | 1 | 10,5 |
| McGinnigle, 2017 | 1,5 | 0,5 | N/A | 3 | N/A | 1 | 1 | 1 | 8 |
| Mullins, 2020 | 2 | 1,5 | N/A | 3 | N/A | 2 | 1 | 1 | 10,5 |
| Munoz-Velandia, 2019 | 1 | 1,5 | N/A | 3 | N/A | 2 | 1 | 1 | 9,5 |
| Nason, 2012 | 1 | 1,5 | N/A | 3 | N/A | 2 | 1 | 1 | 9,5 |
| Ni hlci, 2020 | 1,5 | 0,5 | N/A | 3 | N/A | 1 | 1 | 1 | 8 |
| Nocera, 2019 | 2 | 1,5 | N/A | 3 | N/A | 2 | 1 | 1 | 10,5 |
| O'kelly, 2017 | 1 | 1,5 | N/A | 3 | N/A | 2 | 1 | 1 | 9,5 |
| Ordonez-Gutierrez, 2020 | 1 | 1,5 | N/A | 3 | N/A | 2 | 1 | 1 | 9,5 |
| Ortega, 2017 | 1 | 1,5 | N/A | 3 | N/A | 2 | 1 | 1 | 9,5 |
| Patino-Hernandez | 1 | 1,5 | N/A | 3 | N/A | 2 | 1 | 1 | 9,5 |
| Rosenkrantz, 2017 | 1 | 1,5 | N/A | 3 | N/A | 2 | 1 | 1 | 9,5 |
| Smith, 2019 | 1 | 0,5 | N/A | 3 | N/A | 2 | 1 | 1 | 8,5 |
| Thangasamy, 2014 | 1,5 | 0,5 | N/A | 3 | N/A | 1 | 1 | 1 | 8 |
| Thoma, 2018 | 2 | 0,5 | N/A | 3 | N/A | 2 | 1 | 1 | 9,5 |
| Tonia, 2016 | 3 | 0,5 | N/A | 3 | N/A | 2 | 1 | 1 | 10,5 |
| Truegar, 2018 | 1,5 | 0,5 | N/A | 3 | N/A | 2 | 1 | 1 | 9 |
| Wadhwa, 2017 | 1 | 0,5 | N/A | 3 | N/A | 2 | 1 | 1 | 8,5 |
| Wang, 2017 | 1 | 1,5 | N/A | 3 | N/A | 2 | 1 | 1 | 9,5 |
| Widmer, 2019 | 3 | 0,5 | N/A | 3 | N/A | 2 | 1 | 1 | 10,5 |
| Wong, 2018 | 1 | 1,5 | N/A | 3 | N/A | 2 | 1 | 1 | 9,5 |
| Zhou, 2018 | 1 | 1,5 | N/A | 3 | N/A | 2 | 1 | 1 | 9,5 |

| *Question 2.2 is n/a because data comes from database |
| --- |
| *Question 4 is not applicable because no psychological construct is measured |
